# The Story of the London Fever Hospital

**Published:** 1888-11-03

**Authors:** 


### The Story of the London Fever Hospital.

By One of the Governors.

In the year of grace 1802, there was opened at No. 2, Constitution Road, Gray's Inn Road, a "House of Recovery" for the reception of patients suffering from the then known forms of contagious fever other than small-pox. This tiny hospital, holding in all but fifteen beds, besides three for nurses, was the parent of the London Fever Hospital at Liverpool Road.

The earliest known instance of any means being taken to isolate fever patients was at Cheltenham, where fever wards were established by Dr. Haygarth in 1783. In Manchester, too, about the same time, the need for separating the sick from the healthy was, to some small extent, carried to practical effect. But, with these and a few other exceptions, no effort was made to stay the march of the terrible diseases which laid waste the overcrowded and unhealthy courts and alleys of our great towns.

In March, 1800, a report was published by Dr. Willan which showed the enormous rate of mortality from fever among the poor, and pointed to overcrowding, dark cellars used as dwellings, attics with the windows stopped up to escape window-tax, and general habits of uncleanliness, as the exciting causes.

The Society for Bettering the Condition of the Poor now took up the work, and at a public meeting held on May Day, 1801, it was resolved to establish a House of Recovery for the sick, and to organize a system of cleansing and white-washing houses and rooms where fever had broken out, and of lending linen and other things to the patients at their own homes.

At the time when the House of Recovery was set on foot Constitution Row was within sight of green fields. To quote the words of an article published in *All the Year Round*, then edited by Charles Dickens: "The Blue Lion Inn, on one side of St. Andrew's burial-ground, and the Welsh Charity School, standing in grounds of its own, on the other, were the only buildings in the green lane between Guildford Street and Constitution Row. From the bottom of Acton Street, then a very short street, which ran out of Constitution Row on the right hand side, and led into fields, one might walk over fields, without passing a single house, to Sadlers' Wells, and thence on across the thin and airy slip of the houses of Islington; but otherwise still over fields without touching a house, except the thinly-scattered line of detached villas in the City Gardens, all the way to Hoxton" ("Growth of a Hospital," *All the Year Round*, August 10th, 1861).

Such was the situation of the first Fever Hospital in London. The staff of the new hospital consisted of two physicians, called extraordinary, whose duties would appear to have been consultative, a visiting physician, an apothecary, a matron, nurses, and a maid-of-all-work. The name of the first visiting physician, Dr. Murray, is recorded in the minute-book as that of the earliest victim to the disease he gave his life to grapple with. Stricken with disease caught in some fever-den in Stonecutter's Alley, Lincoln's Inn Fields, he died within the first year of the opening of the hospital. The committee, in token of their appreciation of his services, presented to his mother a silver urn, the inscription on which is duly recorded in the minute-book.

Some of the parish authorities now began to see the value of the new institution, and suggestions were made for the payment by them to the institution of a certain fixed sum for every patient sent in by the former. But lack of funds and space threatened to cripple, if not to stop the work of the young hospital. Mainly through the exertions of the great William Wilberforce, a Parliamentary grant of three thousand pounds was made to the parent Society for Bettering the Condition of the Poor, to be held in trust for the purposes of the hospital.

The committee now began to cast about for larger and more convenient premises; and a curious fact which is recorded in connexion with the house in Constitution Row is noteworthy, as showing the difficulties that even then beset the efforts of those interesting themselves in the cause of disease prevention. The landlord of the house alleged that his garden adjoining had become useless to him, because it was supposed that the air was contaminated by reason of its nearness to the House of Recovery. The committee at once rented the garden at eight pounds a year, and expected to recoup themselves by the value of the produce!

In 1811 an estate at Coldbath Fields, including the Cold Bath itself, an old and sometime famous mineral spring with a reputation for the cure of scrofulous complaints, was bought by the institution for £3,630, with the intention of devoting a part of it to the uses of a House of Recovery. Opposition to this scheme was at once encountered from the Clerkenwell Vestry, and eventually an offer of the old Small-pox Hospital, at King's Cross, to hand over for a moderate sum the western wing of their building for the reception of fever, the House of Recovery was established and remained there until, along with the Small-pox Hospital, the great dust-heap, and the camera obscura, it was swept away to make room for the Great Northern Railway terminus.

From this time until 1849, when the new hospital in Liverpool-road was opened, the House of Recovery continued its useful work as the only fever hospital in the metropolis.

Fevers were in those days, especially typhus, endemic in the poorer parts of London, and though the records of the hospital tell of recurring outbreaks of greater fury than usual, there was always a steady demand on the resources of the House of Recovery that kept the efforts of the committee in constant work.

In 1843 the earliest of the printed reports to which access is to be had, there was an epidemic of "continued fever," and many patients had to be refused admission for lack of room. The cause of this outbreak is attributed to the influx of a large number of agricultural labourers and mechanics from the country in search of work in London. Among twenty-nine servants of the hospital attacked with fever three "barbers" are recorded, one of whom insisted upon going to his own home instead of being nursed in the hospital, and so infected nine other persons. Year after year the Committee have the same tale to tell of typhus spread and cultivated in the overcrowded dens of London. In 1846 a peculiarly bad case is recorded, which is worth notice as showing the sort of thing that was possible in a workhouse of that day. The hospital it appears was never without patients from Marlborough House, Peckham, the then workhouse for the City of London. It was stated to be the most easily accessible shelter for the houseless poor of London, and was therefore filled to excess every night, but on certain occasions, such as the end of harvest and the hopping season, it was specially crowded. At these times as many as a hundred men were crowded into one room 33 ft. 9 in. by 20 ft. and 7 ft. high in the centre, the roof sloping down to 2 ft. at the sides. In this room with an average of 6·75 square feet and 30·38 cubic feet per head, the only means of light and ventilation were two windows 18 inches square.

The new hospital in the Liverpool Road was erected from the designs of the late Mr. Charles Fowler, at a cost of £18,357, including the land and professional charges. There were two large double wards for ordinary patients, with smaller wards for convalescent patients, and some single rooms for noisy patients attached. In addition to these were four small wards destined for the reception of patients able to pay something towards their maintenance, and two wards on the upper floor for convalescent cases. The total accommodation for patients was 182 beds.

The planning of the new hospital was noteworthy for very large cubic space allotted to each patient—2,000 ft. per head. The amount finally determined upon was at that time far in excess of any existing hospital. The construction of the wards too was a notable improvement upon the then known forms. At the London Hospital, and also at St. Bartholomew's, double wards were then as they still are inexistence. They consisted of two distinct wards side by side, with small openings from one side into the other, the division wall being for the most part a solid partition. At the London Fever Hospital the division wall was reduced to its smallest possible dimensions, and the openings made as large as possible, the result being that the wards were far more airy and better ventilated than they would have been had existing models been closely followed. To this day these wards, notwithstanding the fact that they are designed on a principle now discarded, are models of cheerfulness and thorough ventilability.

Great pains was taken both by the committee and their professional advisers to render the ventilation of the new hospital as perfect as possible, and to this end the well-known Dr. Arnott was called into council, and with his aid a system of artificial ventilation was devised. The method adopted was that of a large fan propelled by steam, which could be made either to drive the air into the wards or to draw it out. The fan occupied a central position between the male and female sides, and carefully constructed shafts led to each ward. The system with its apparatus has long been discarded in favour of the more efficient and reliable one of open windows; but the pains and skill brought to bear upon the subject merit our respectful recognition as an earnest and thoughtful attempt to grapple with a most important problem.

Another point about the original design of the hospital is also of peculiar interest. The system of drainage, though naturally not what would be approved in these days, was yet in many respects most efficiently contrived; and when it is considered that the main drain of the hospital, as originally laid down under Mr. Fowler's direction, is to this day doing its work in the most efficient manner; it will be conceded that this part of the work will bear favourable comparison with many buildings of much later date.

To trace the history of the hospital from the time of the opening of the new buildings to the time when, by the opening of the Asylums Board Hospitals, the pauper patients were provided for at the expense of the rates, and the Fever Hospital was thus relieved of a large class of patients, would be in effect to tell the story of ever recurring outbreaks of fever in London. A story which would be rich in lessons of the evils of overcrowding, of dark, dirty, and unhealthy houses, and of insanitary conditions generally. And a story too, which would tell of the incalculable good done by the prompt isolation of patients, many of whom, if left in their own homes, would have formed foci from which contagion to unknown extent would have spread to their fellow-men.

When the Asylums Board had provided hospital accommodation for the pauper class, the committee of the Fever Hospital decided to take a new departure, and provide accommodation of a better kind for those able and willing to pay for it, and accordingly, in 1871, four wards for each sex were formed, and set apart as private wards. The charge for these wards, which are for one patient each, was fixed, and still remains, at three guineas a-week, which is an inclusive one. At the same time the charge for patients in the general wards was fixed at two guineas for the whole period of stay in hospital, a fee which has since been raised to three guineas.

At the same time that these alterations were made the two large double wards, with the convalescent wards attached, which had hitherto been used for typhus or relapsing fever, were assigned to scarlet fever patients, and the upper wards were devoted to typhoid. The wing which had been built in 1866 to meet the demands made upon the hospital by an epidemic of cholera, and which had been partially disused, was in 1881 altered to form two wards for typhoid cases. The erection of a small block of isolation rooms for doubtful cases in 1883, and of a detached building containing the secretary's offices, complete the record of additions and alterations to the original building.

The roll of medical officers and physicians of the London Fever Hospital contains many names whose owners have been of high eminence in their profession; but it will not be deemed invidious to single out two whose services to the cause of the study of fevers are historical. Sir William Jenner, to whose researches we owe the definition of typhus and typhoid as two separate diseases, was assistant physician to the hospital from 1855 to 1861; and Dr. Charles Murchison, whose name is inseparably bound up with the cause of epidemiology was successively assistant physician, physician, and consulting physician, his connexion with the hospital extending from 1856 to his death in 1879.

The work of the London Fever Hospital has always been in the past, and must always be in the future, primarily a work of prevention; though the curative function is an almost equally important part of the work. As a preventive agent it provides for the heads of large business establishments, schools, hotels, and private families a ready means of isolating an initial case of disease, and so removing a possible focus of disease from infectible surroundings. Viewed in this light, it is scarcely possible to overstate the value of the services such an institution renders to society at large; and when it is remembered that although in the course of years the hospital has gradually become surrounded by a densely-populated neighbourhood, yet no single case has ever occurred of disease spreading from within its walls to the outer world, the value of the work done becomes all the more remarkable.

